# Multivariate Statistical Approach for the Discrimination of Honey Samples from Galicia (NW Spain) Using Physicochemical and Pollen Parameters

**DOI:** 10.3390/foods12071493

**Published:** 2023-04-01

**Authors:** Olga Escuredo, María Shantal Rodríguez-Flores, Montserrat Míguez, María Carmen Seijo

**Affiliations:** 1 Department of Vegetal Biology and Soil Sciences, Faculty of Sciences, University of Vigo, As Lagoas, 32004 Ourense, Spain; 2 Department Analytical and Food Chemistry, Faculty of Sciences, University of Vigo, As Lagoas, 32004 Ourense, Spain

**Keywords:** Spain, honey, melissopalynology, quality parameters, polyphenols, color, chemometrics

## Abstract

Raw honey is a food with a close relation to the territory in which it is produced because of factors such as soil conditions, weather patterns, and plant communities living in the area together. Furthermore, beekeeping management affects the properties of honey. Protected Geographical Indication *Miel de Galicia* protects the honey produced in Galicia (Northwest Spain). Various types of honeys (362 samples) from this geographical area were analyzed using chemometric techniques. Principal component analysis was favorable to analyzing the physicochemical and pollen variables with the greatest weight in the differentiation of honey. The linear discriminant analysis correctly classified 89.8% of the samples according to the botanical origin using main pollen spectra and physicochemical attributes (moisture, pH, electrical conductivity, diastase content, phenols, flavonoids, and color). Regarding unifloral honey, blackberry, eucalyptus, and heather honeys were correctly grouped, while five chestnut honeys and fourteen samples of honeydew honeys were misclassified. The chestnut and honeydew honeys have similar physicochemical properties and frequently similar pollen spectra profiles complicating the differentiation. Experimental evidence suggests the potential of multivariate statistics in the characterization of honey of the same geographical origin. Therefore, the classification results were good, with electrical conductivity, total phenol content, total flavonoid content and dominant pollens *Eucalyptus*, *Erica*, *Rubus* and *Castanea sativa* as the variables of higher importance in the differentiation of botanical origin of honeys.

## 1. Introduction

Honey is considered a natural and animal product because it is produced by honey bees, but the main sources are plant secretions such as nectar (known as a blossom or floral honey) or honeydew harvested on the living parts of plants (honeydew honey). In the hive, the bees transform this mixture of different substances composed mainly of sugars, water, proteins, and other compounds such as enzymes, organic acids, vitamins, and phenols in honey [1,2,3]. Glucose and fructose are the carbohydrates present in the greatest amount and contribute mainly to the energy value and physical characteristics of honey. Other minor constituents of honey are proteins, enzymes (such as diastase, invertase, and glucose-oxidase), amino acids and organic acids (proline and gluconic acid, as the most abundant, respectively), vitamins (mainly vitamin C and group B), and minerals (K, Ca, Na and P, among others) [1,4]. Honey contains diverse phytochemical substances biosynthesized by plants that have important antioxidant activity [5]. Among them, phenol acids and flavonoids play a significant role in their bio-functional properties, as well as in their antioxidant and anti-inflammatory activities [6,7]. Some findings also confirmed the contribution of some aliphatic acids from royal jelly in the antibacterial action of honey [8]. Hence, honey is recognized as one of the most popular natural functional foods, which is attributed to many healthy properties. Therefore, this bee product is used in a wide variety of nutritional and medicinal products and cosmetic applications [2,3,9]. Moreover, it has been used as a food ingredient in interesting culinary arts for its attractive physical and organoleptic qualities [4].

The quantitative variability of each chemical compound in the composition of bee products defines the versatility of its physical, sensory, and functional characteristics [6]. The chemical constituents of honey, as well as the viscosity of its texture, are variable and primarily depend on the floral source, but some external factors, such as seasonal and environmental factors and the processing method, are also influential [1,7,10,11]. One of the most affected properties is color because the nectar origin and its composition contribute to the wide diversity perceptions, from almost water white to black, that can be seen according to the honey type. Thus, some compounds are related to a darker hue of honey, and this is the case of minerals, phenol acids and flavonoids [12,13,14]. The importance of the color of honey lies in consumer preferences and the choice for one type of honey or another, and consequently, it is closely linked to the commercial value of the product. Generally, lighter honeys fetch higher prices on the market, but these preferences vary by country and region. In recent years, dark honey types such as honeydew honey have been gaining the market in countries such as Germany, Switzerland, Greece, and Turkey [4], as well as in Spain. Hence, the importance of their study relating this honey type with the area of production and the source of honeydew.

The relationship of the honey composition with its origin is a relevant issue since it is the tool used to assess the authenticity of honey [7,9]. Several researchers showed differences in the characteristics of honey according to their floral and geographical origins [1,11,14,15,16,17,18]. In Spain, the production of a wide range of honeys with different physicochemical and antioxidant characteristics has been reported depending on the production area [1,12,14,19,20,21,22], with a distinguishable unifloral gap between the north and the south of the country. The most representative unifloral honeys in south-central Spain are orange blossom honey (*Citrus aurantium*), holm oak or oak honey (*Quercus ilex*), lavender honey (*Lavandula angustifolia*), sunflower honey (*Helianthus annuus*), rosemary honey (*Rosmarinus officinalis*), and thyme honey (*Thymus*). In the northern area of the Iberian Peninsula, chestnut honey (*Castanea sativa*), blackberry honey (*Rubus*), eucalyptus honey (*Eucalyptus*), and honeydew honey (*Quercus pyrenaica*) are produced. Heather honey obtained from a different *Erica* species or from *Calluna vulgaris* can also be found throughout the Spanish territory.

The first honey quality criteria observed by consumers are the color and the density or degree of crystallization. Therefore, they are the determining parameters in the selection of the purchase. In this sense, beekeepers, retailers, consumers, and food safety regulatory offices are interested in knowing the quality of honey based on botanical origin and its particular physicochemical properties [1]. Unfortunately, honey products are often adulterated and mislabeled. Honey can be adulterated with cheaper and poorer quality honey, sugar solutions or syrups with high fructose content. The composition of honey can also be manipulated by feeding the bees with sugars and syrups, producing artificial honey [23]. Sometimes, the labelling is changed to a specific botanical or geographical origin to increase the commercial value in the market. For this reason, the adulteration of honey has become a global problem, which in addition to affecting the consumer, undermines the efforts of many beekeepers in the struggle to produce excellent products with qualities worthy of recognition. Honey authentication, regarding its genuine botanical and geographical origins, as well as the detection of any adulteration, is essential in order to protect consumer health and avoid unfair competition that could create a destabilized market [9].

The Protected Geographical Indication (PGI) *Miel de Galicia* is a designation of origin registered in the European Union since 2007. It covered a territory of about 29,500 km^2^, with traditional apiculture mainly based on non-migratory apiaries, producing more than 500 t of honey under the label [24]. This quality designation is crucial for the development of beekeeping in the area and the increase of the economic value of honey. This is also in addition to the close contribution to the maintenance of biodiversity and rural environments. Therefore, this type of research can collaborate with the strategy of linking honey and territory throughout the botanical origin and key physicochemical parameters. The present study is the first work that includes such a large number of samples from the same geographical territory for this purpose. There are several investigations that typify the physicochemical qualities of European honeys depending on the botanical origin [1,4,15,16,18,19,20,22,25,26,27,28,29]. However, the reliability of the results depends on the large extent of the number of samples treated with similar characteristics, which allows the successful valorization of unifloral honey.

Currently, tools that handle a large set of data and resulting variables are being sought to identify common physicochemical patterns in the honey. Multivariate analysis has the advantage of extracting information from complex data sets using mathematical and statistical techniques. Among them, the principal component analysis (PCA), the cluster analysis (CA) or the linear discriminant analysis (LDA) offers the possibility of analyzing a complex food matrix such as honey and making a possible classification by botanical origin [4,18,20,21,22,27,28,29].

The aim of this study was to characterize and classify honeys collected in a specific Atlantic region according to their botanical origin. Firstly, a description of the palynological and physicochemical characteristics of the set of honey samples from the geographic area is detailed. Secondly, it is intended to classify honey regarding the botanical origin using a multivariate statistical treatment applied to pollen variables and physicochemical quality parameters such as color, total phenol, and flavonoid content. The similarities and the weight of the variables analyzed according to different statistical treatments are evaluated for the subsequent interpretation of the classification rate of the samples according to their botanical origin.

## 2. Materials and Methods

### 2.1. Geographical Origin of Honey Samples

The study was carried out with 362 samples of honey collected in different localities of Galicia (Northwest Spain). Sampling was carried out in collaboration with beekeepers and technicians of beekeeping associations as well as PGI *Miel de Galicia* during eight harvest seasons. The samples were deposited in glass containers and transported to the laboratory in the Faculty of Sciences (Ourense, Spain). The quality parameters were carried out upon arrival at the laboratory, and the remaining samples were stored frozen until further analytical determinations. The analytical determinations were performed in duplicate.

The honeys were collected without classification of their botanical origin. The palynological analysis and the physicochemical parameters (detailed below) were the basis for classifying the honey samples according to their botanical origin and subsequent statistical treatment.

### 2.2. Melissopalynological Analysis of Honey

The melissopalynological analysis of honey was performed by extracting the sediment from the samples. A total of ten grams of sample were dissolved in 50 mL of water until it had been completely dissolved. After this, the solution was centrifuged at 4500 rpm (3373 *g*) twice, for 10 min and 5 min, respectively [5]. Then, the supernatant was discarded. For the quantitative analysis, the volume of the sediment was completed until 5 mL, after stirring 10 μL of the sediment, was deposited in a slide over a heat plate. When the aliquot dried up, a drop of glycerol–gelatine stained with fuchsine was added to the sample and then covered with the coverslip for counting. In the case of qualitative analysis, new centrifugation at the same conditions was performed, and the supernatant was discarded. An aliquot of 100 μL of the sediment was deposited on the slide and covered as commented before. The procedure was carried out by duplicate. The identification and counting of the pollen grains were performed in both subsamples under optical microscopy (Olympus BX50 microscope, Olympus Corp., Tokyo, Japan). The minimum number of pollen grains counted for qualitative analysis was 700 pollen grains, and the results of each pollen were expressed as percentage over the total number of pollen grains counted. For quantitative analysis, all the pollen grains in both subsamples were counted, and results were expressed as number of pollen grains by gram of honey.

### 2.3. Determination of Quality Parameters: Moisture, pH and Electrical Conductivity

The moisture content of the honey was determined with an ABBE URA-2WAJ-325 digital refractometer (Auxilab S.L., Navarra, Spain) using the refractive index values from the Chataway table at 20 °C. The pH was measured directly on honey sample solutions in bi-distilled water (0.2 g/mL) using a pH meter (Crison micro pH 2001; Crison Instruments S.A., Barcelona, Spain). The electrical conductivity (EC) was measured on the same honey solution with a portable conductivity meter (Knick Portamess 913 Conductivity, Beuckestr, Berlin), expressing the results as mS/cm. Schade method was used to determine diastase activity of honeys [30]. It was calculated based on the hydrolysis rate of the starch solution by α-amylase present in a honey buffer solution at 40 °C. The amount of converted starch in the honey solution was analyzed using a UV-VIs spectrophotometer (Jenway 6305 UV-Visible spectrophotometer, Staffordshire, UK) at an absorbance of 660 nm. Measurements were taken at various time intervals until an absorbance of less than 0.235 was reached. Finally, the diastase activity was calculated as the diastase number or grams of hydrolyzed starch per hour per 100 g of honey.

### 2.4. Determination of Color

The color of the honey was determined with a HANNA Honey Color C221 colorimeter (HANNA C221 Honey Color Analyzer, Rhode Island, RI, USA). The fluidity of the honeys was previously treated to the measurement of the color for the correct reading. The treatment consisted of heating the slightly fluid or crystallized honeys no more than 45 °C in a thermostatic bath [13]. After a short break (for the total elimination of possible bubbles), approximately 4 mL of honey sample was introduced into a smooth plastic vial. Glycerin was used to calibrate the instrument. Finally, the color was expressed in mm, according to Pfund scale.

### 2.5. Determination of Total Phenol and Flavonoid Concentration

The total phenol content (TPC) and the total flavonoid content (TFC) were determined by spectrophotometric techniques according to reference methods proposed by Singleton et al. [31] and Arvouet-Grand et al. [32], respectively. For the determination of TPC, solutions of honey samples (0.1 g/mL) were prepared. These solutions were mixed with Folin–Ciocalteu reagent and calcium carbonate solutions, and the absorbance at 765 nm using a UV-Vis spectrophotometer (Jenway 6305, Staffordshire, UK) was measured. A calibration curve was obtained using gallic acid solutions (0.01–0.50 mg/mL) as a reference standard to quantify TPC. The methodology for the determination of TFC starts with preparing the honey sample solutions (0.33 g/mL). Then, a volume of aluminum chloride solution was added, and the absorbance was measured against a blank at 425 nm with a UV-Vis spectrophotometer (Jenway 6305, Staffordshire, UK). For the quantification of flavonoids, a curve with quercetin (0.002–0.01 mg/mL) as a reference standard was used. Finally, TPC and TFC were expressed in mg/100 g honey.

### 2.6. Statistical Analysis

The statistical treatments were carried out with SPSS Statistic 23.0 (IBM SPSS Statistics, Armonk, NY, USA) and Statgraphics Centurion 17.0 for Windows (Statgraphics Technologies, Inc., The Plains, VA, USA). Multivariate techniques were applied as association tools, searching for common patterns and relationships in masses of data. Principal component analysis (PCA) and cluster analysis (CA) were performed to reduce the amplitude of the data matrix and to establish significant relationships between palynological and physicochemical variables of honey. The statistical results were represented graphically with precise representations that integrate the interrelation of the significant elements between the main pollen types and the physicochemical parameters. Based on the pollen profile and physicochemical parameters, the honeys into four unifloral honey groups were classified: chestnut, blackberry, heather, and eucalyptus (with 52, 56, 36 and 33 samples, respectively), a honeydew honey group (with 53 samples) and the multifloral group (with 132 samples).

The classification of honey samples was checked through linear discriminant analysis (LDA) based on certain similarities in their physicochemical and botanical characteristics. Therefore, LDA was tested to quantify the probability of belonging to one type of honey or another. Finally, with the objective of comparing the groups of samples classified according to the results of the multivariate treatment, an analysis of variance (ANOVA) was performed using the Bonferroni test (*p* < 0.05).

## 3. Results

### 3.1. Representation of Botanical Diversity in Galician Honeys

The variability of families and pollen types identified in all the samples are classified as 52 and 111, respectively. The pollen types with the highest representation in the honey samples were *Rubus*, *Castanea sativa*, *Cytisus* type, *Erica*, *Eucalyptus*, *Trifolium* type, *Quercus*, and *Echium* (in more than 60% of the honeys) (Table 1). 

The pollen grains with a higher mean percentage were *Castanea sativa* (42.9%), *Rubus* (23.2%), *Eucalyptus* (13.4%), *Erica* (7.9%), and *Cytisus* type (5.3%). *Eucalyptus*, *Castanea sativa*, and *Rubus* had a maximum value above 90%. Finally, the diversity in the pollen profile of the honey produced in Galicia was reflected in the quantitative analysis performed in the sediment of the samples. The counted number of pollen grains had an average value of 20,879 grains/g, with a wide standard deviation of 21,398 grains/g.

### 3.2. Physicochemical Characteristics of Honeys

Descriptive analyses for the results of physicochemical parameters and color are shown in Table 2. The moisture content of the studied honeys showed a mean value of 17.74%, with a range between 14.4% and 21.2%. The minimum values of pH and EC were 3.29 and 0.22 ms/cm, and the maximum values were 5.14 and 1.65 ms/cm, respectively. In terms of mean values, 4.24 for pH and 0.76 mS/cm for EC were obtained. Diastase content had a low mean value (21.31), with a range between 6.14 and 44.04. The mean values for TPC and TFC were 116.43 mg/100 g and 6.72 mg/100 g, respectively. However, these compounds presented a great variability in the set of samples, with a range between 33.91 and 254.5 mg/100 g for TPC and 1.28 and 16.7 mg/100 g for TFC. Finally, the honeys ranged from an amber color to dark color, from 150 mm to 36 mm on the Pfund scale.

### 3.3. Distribution of Honeys According to Botanical Origin, Physicochemical Parameters and Multivariate Classification Techniques

PCA was the multivariate technique used to simplify the large database matrix, and at the same time, it allowed us to show the relationships between the physicochemical and pollen variables. A total of four components were extracted that explain 76.14% of the variability of the original data (Table 3). The first two components accounted for more than 50% of the data variability. The variables with the greatest weight in the first component of PCA were pollen variables, such as *Eucalyptus* and *Castanea sativa*, and the physicochemical variables EC, TPC, and TFC. In the second component, *Erica*, *Rubus*, moisture content, color, and pH had the highest weight in the analysis.

**Figure 1:**
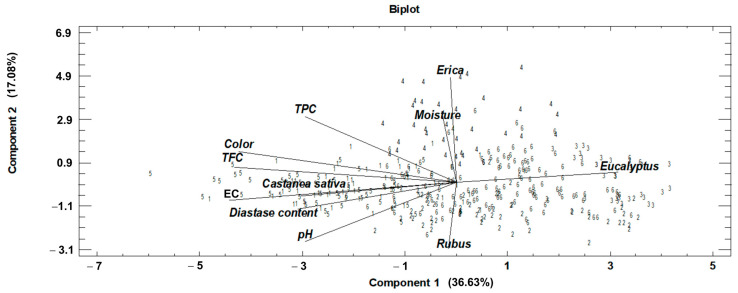
Biplot of the first two components and distribution of honey samples extracted by PCA. EC: electrical conductivity; TPC: total phenol content; TFC: total flavonoid content. Numerical nomenclature of honeys according to botanical origin: 1, chestnut; 2, blackberry; 3, eucalyptus; 4, heather; 5, honeydew; 6, multifloral.

The graphical representation of these two components shows the distribution of the variables included in the analysis and the distribution of the honey samples according to them (Figure 1). The honeys were categorized by a number from 1 to 6 due to their botanical origin (1: chestnut, 2: blackberry, 3: eucalyptus, 4: heather, 5: honeydew, 6: multifloral) for better visualization of the distribution. Thus, the honeys with a high percentage of *Castanea sativa*, high EC, pH, higher enzyme content and higher TFC were located on the left side of the quadrant. TPC variable was located between the honeys with a high content of *Castanea sativa* and *Erica*. At the same time, the samples with higher *Erica* pollen were those with higher moisture content. The *Rubus* pollen variable is placed on the opposite side of *Erica*. Finally, a group of samples was located together with the *Eucalyptus* variable on the opposite side of *Castanea sativa* pollen, EC, pH, color, and diastase content. Therefore, the honeys located on the right side of the quadrant are characterized by being the clearest, with lower EC and pH, lower diastase content, and lower TPC and TFC, mainly honeys which predominate *Eucalyptus* pollen. The darker honeys had the highest electrical conductivity, flavonoid content, and frequently large percentage of *Castanea* pollen.

**Figure 2:**
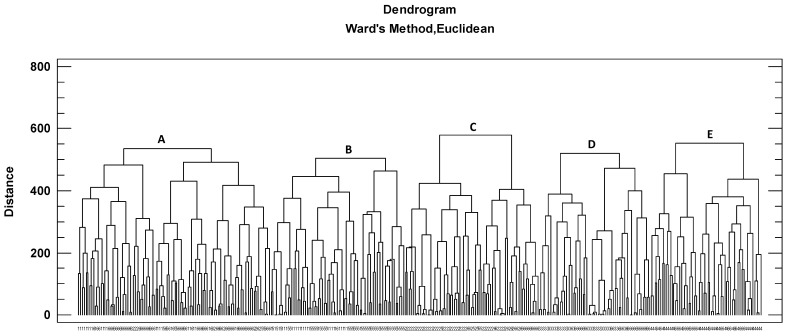
Honey samples distributed in five groups (cluster A, B, C, D and E) according to CA. Numerical nomenclature of honeys according to botanical origin: 1, chestnut; 2, blackberry; 3, eucalyptus; 4, heather; 5, honeydew; 6, multifloral.

CA was applied to analyze the groups of homogeneous honeys based on the physicochemical variables and main pollens (Figure 2). The results showed a good grouping of honeys of heather and eucalyptus (clusters E and D, respectively). Cluster C grouped blackberry samples with some honeydew and multifloral honeys. Cluster B included chestnut honeys and some honeydew honeys. Finally, cluster A (the largest group of samples, with 102 honeys) included samples of chestnut, honeydew, blackberry and multifloral.

### 3.4. Classification Rate of Honeys According to Botanical Origin

Based on the results of the pollen profile and the physicochemical parameters, 177 honeys were classified as unifloral honeys (chestnut, blackberry, eucalyptus, and heather), 53 as honeydew honeys and the remaining samples were grouped as multifloral honey. This previous classification was considered in the LDA for the discrimination of honeys based on botanical origin. LDA results were satisfactory, with five discriminant functions (Table 4). The first three functions optimally separated the samples with a percentage of the relative variance of the data higher than 90% and a canonical correlation greater than 0.80 with eigenvalue values above 2.2. Results of LDA showed that two statistically significant discriminant functions are formed (Wilks Lambda = 0.01, Chi-Square = 1777.24, degrees of freedom = 55, *p* < 0.05 for the first function, and Wilks Lambda = 0.03, Chi-Square = 1211.01, degrees of freedom = 40, *p* < 0.05 for the second, respectively). Low values of Wilks Lambda (close to 0) indicated high discriminant power because the mean of the explanatory variables included in the analysis is different between the groups (honey type), mainly in the first two discriminant functions.

**Figure 3:**
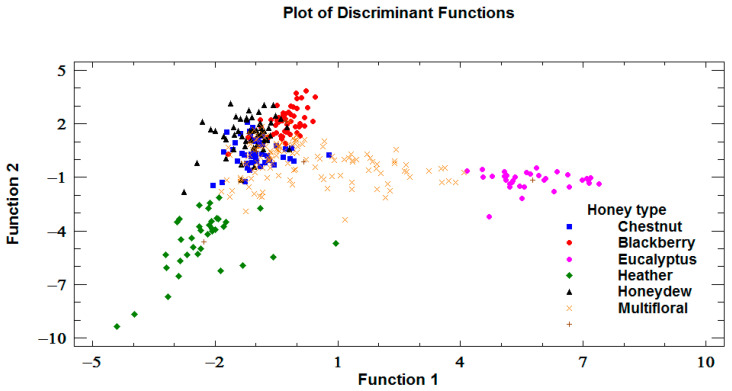
Plot of the representation of the first two discriminant functions and distribution of samples by honey type.

The classification of honeys by LDA resulted in satisfactory, correctly classifying 89.8% of all the samples. Blackberry, eucalyptus, and heather honeys were properly classified (100%). However, five samples of chestnut honeys (9.6%), 14 samples of honeydew honeys (22.4%) and 18 samples of multifloral honeys (13.6%) were misclassified (Table 5). Figure 3 shows the distribution of the samples by honey type, with a clear differentiation of heather and eucalyptus honeys with respect to the others, which is due to their different physicochemical qualities. The first two functions extracted by LDA are dominated by the variables: *Eucalyptus* (with a standardized discriminant function coefficient of 1.07), *Erica* (−0.88), *Rubus* (0.37), color (0.32), and TPC (0.31).

### 3.5. Physicochemical and Melissopalynological Characterization of Unifloral and Honeydew Honeys

The characterization of the unifloral, honeydew and multifloral honeys according to the main palynological and physicochemical characteristics through the multivariate treatment are detailed in Table 6. An analysis of variance (ANOVA) according to the Bonferroni test was applied to evaluate the significant differences and similarities by honey type. The most outstanding characteristics of honey type are detailed below.

Based on these results, chestnut honeys are characterized by a mean percentage of *Castanea sativa* of 76.8% (significantly different from the other characterized honey types, *p* < 0.05) and with a confidence limit of 95% above 70%. The counted pollen grains were significantly higher in average terms than eucalyptus, heather and multifloral honeys. EC and pH of chestnut honeys had significantly higher mean values (1.02 mS/cm and 4.5, respectively) with respect to unifloral honeys (*p* < 0.05) but similar to honeydew honey (1.14 mS/cm and 4.5, respectively). The diastase content was intermediate, with a mean value significantly lower (23.5) than honeydew honey (29.4) and significantly higher than eucalyptus honey (14.6) (*p* < 0.05). Regarding the quantification of TPC and TFC, the chestnut honeys were characterized by a significantly higher content with respect to the other unifloral honeys (*p* < 0.05), except for heather honey (with similar mean values) and honeydew honey that have higher mean values (*p* < 0.05). Finally, the color of this group of honeys is dark, with a mean value of 128 mm, similar to heather and honeydew honeys (117 and 142 mm, respectively).

The blackberry honeys were characterized by a mean percentage of *Rubus* of 56.7% and a lower limit (95% confidence level) above 53%. Some physicochemical characteristics that differentiate them from other honeys of the area were pH, which is significantly higher (4.3, *p* < 0.05) and TFC, with a significantly lower mean value (6.1 mg/100 g, *p* < 0.05). In addition, TPC was significantly lower than chestnut, heather and honeydew honeys (*p* < 0.05). On the other hand, the EC of blackberry honey (0.69 mS/cm) was significantly lower with respect to chestnut and honeydew honeys (mean values above 1.0 mS/cm) and significantly higher with eucalyptus honey (0.51 mS/cm).

The eucalyptus honeys were characterized by a mean value of 73.8% for *Eucalyptus* pollen, with a minimum value at a 95% confidence level of 70.1%. These are honeys with the lightest color (77 mm), lower pH, EC, and TFC, and are significantly different to the other groups of honeys studied (*p* < 0.05). pH was similar to heather honeys (4.1) but significantly lower than the other unifloral honeys (*p* < 0.05).

The mean percentage of *Erica* in the heather honeys was 36.7%, with confidence limits between 33.1% and 40.3%. The counted pollen grains were significantly lower than chestnut and blackberry honeys (*p* < 0.05). Despite having a lower percentage of dominant pollen representation than other unifloral honeys, it has well-marked physicochemical properties. It highlights the color, with a significantly higher average value (117 mm) with respect to eucalyptus and blackberry and significantly lower with honeydew honeys (*p* < 0.05). TPC and TFC presented significantly higher mean values (143.1 mg/100 g and 7.3 mg/100 g, respectively), similar to chestnut honeys (*p* < 0.05).

The honeydew honey had more pollen diversity, with mean values significantly different in *Rubus* and *Castanea sativa* compared to the other types of unifloral honey (*p* < 0.05), but with lower values. This type of honey presented physicochemical parameters statistically different to eucalyptus, heather, and blackberry honey for pH, EC, and color (with significantly higher values, *p* < 0.05). However, the values were similar to those of chestnut honey, hence the possibility of finding honeys based on nectar secretion obtained from chestnut and contributions of honeydew that give particular properties to these samples.

Finally, multifloral honeys were composed of samples with a heterogeneous pollen profile. In most of these honeys, there was not a predominant pollen type in the pollen spectra, or if there were one, it was generally *Castanea sativa*. *Rubus*, *Eucalyptus* or *Cytisus* type usually appeared with values over 10%. Regarding the physicochemical characteristics, they differed in terms of botanical contributions. Although generally, they had lower pH, EC and moisture content and an intermediate enzymatic and polyphenolic content (TPC and TFC). Regarding the color scale, it ranged from dark amber to light amber, with a mean value slightly greater than eucalyptus honey.

## 4. Discussion

Honey, as a valuable natural product of the bee, offers substantial nutritional, therapeutic and medicinal benefits attributed to its botanical origin resulting from its complex chemical composition [2,29]. Some analytical methods are standardized for honey, but an exhaustive analysis of a set of analytes and physical properties is required for its correct characterization [9,23]. Experts from the scientific community and the beekeeping sector emphasized that the labelling with respect to certain botanical or geographical origins cannot be conducted based on a single group of chemical markers but rather on a combination of several [9]. Hence, the characterization of honeys based on their physicochemical characteristics supported by a palynological analysis, sensory analysis, and biologically active compounds contributes to supporting the demand of consumers, regulatory councils, and the beekeeping sector.

Considering the honey types studied from the Northwest of Spain, chestnut, heather, and honeydew honeys had the highest TPC and TFC in comparison to eucalyptus and blackberry honeys. With respect to the quality parameters, chestnut and honeydew honeys had the highest values of EC, pH, diastase, and color by the Pfund scale, unlike heather and eucalyptus honeys which had the lowest EC and color. In previous research on the composition of honeys collected in the Atlantic area of the Iberian Peninsula, the contribution of *Erica* pollen to the content of polyphenolic compounds was reported [1,26], and that derives in a high content of these compounds in unifloral heather honeys, as found in the present study. The physicochemical pattern of Galician chestnut honeys was common to chestnut honeys produced in other countries [25,33], although higher EC, TPC and lower color by CIELab were found for Croatian honeys [27], and lower TPC for Italian chestnut honeys [18]. Chestnut honeys from Tenerife Island are characterized by higher EC and pH than Galician honeys as a consequence of the specific edaphoclimatic characteristics of this territory and the abundance of endemic plants in the Islands [20]. Galician heather honeys were analogous to Portuguese heather honeys [26,33]. In fact, most are obtained from the same species standing out as *Erica umbellata* and *E. arborea* in soil conditions comparable to those of the Galician community. However, Algerian heather honeys showed slightly higher EC, TFC and color measured by the Pfund scale [11,34]. In the case of eucalyptus honey, the predominant specie for Galician honey production is *Eucalyptus globulus*, which flowers in winter and the early spring, in contrast to other European areas where summer production obtained from species such as *E. camaldulensis* is relevant. Concretely, an important production of unifloral eucalyptus honey was documented in Italy, Portugal, and other areas of Spain [25]. The physicochemical properties were similar to these honeys produced in Portugal because of similarities in the ecosystems formed by eucalyptus trees [33]. However, Italian eucalyptus honeys had lower TPC [18] than Galician honeys. In the case of Uruguayan eucalyptus honeys, higher EC, pH, and color were determined [35], while for Algerian eucalyptus honey, similar physicochemical characteristics were reported [34].

Honeydew honey usually presents higher content of bioactive compounds such as phenolic acids, flavonoids, proteins, and amino acids compared to blossom honeys, as documented in honeydew honeys from other geographical origins [2,12,18,27,29,34]. The difficulties in the discrimination of honeydew honey have been referenced because there are various sources of honeydew depending on the plant and the secretion itself by sucking insects or by the plant [17,21,25]. As indicated by Vasić et al. [17], the description of honeydew honeys without specific botanical attribution could explain the variability of physicochemical results in this group of honeys. Currently, in Spain, the types of honeydew honey identified are from holm oak (*Quercus ilex*), green oak (*Quercus pyrenaica*), and other oak (*Quercus* sp.) [21,22,36]. Therefore, in the specific case of this type of honey, a combination of chemical, physical, organoleptic, and statistical data could contribute to its discrimination.

The influence of physical and chemical indicators and the botanical origin in the color of honey is known, contributing to the diversity of its commercial assortment. According to Szabó et al. [37], the color of the honeys is attributed to the predominant plant species in them. However, it is a parameter strongly affected by biogeography because it determines the different plant communities. This justifies that unifloral honeys from the same plant origin present differences in physicochemical properties, as already mentioned for unifloral chestnut honeys produced in Canary Island [20], Croatia [27], or Italia [18]. Each biogeographical area has particular soil conditions, weather patterns, and plant communities that contribute to the differentiation of small nuances in honey. Hence the importance of geographical indications corroborated with the pollen profile in samples. Some specific chemical compounds may be involved in color attributes. This is the case with some phytochemicals, such as flavonoids, which are considered substances with a major effect on chromatic parameters [12,14]. These compounds are present in nectar and honeydew, therefore, are transferred to the hive and become part of the final product as honey [5,10]. In this context, the polyphenol content of dark-colored honeys (such as heather, chestnut or honeydew) [1,4,13,35,38] is usually greater than light samples (such as citrus, eucalyptus or acacia, for example) [1,4,18,23]. The role of polyphenols and flavonoids in plants is not only related to sensory properties such as color or odor or, for example, bitterness; their importance in chemical defense and oxidative processes is a crucial key [39]. Consequently, dark honeys show higher antioxidant activity [1,4,5,15,18,23,38].

At the same time, handling such a large matrix of data requires complex data management. The application of advanced multivariate chemometric techniques contributes to analyzing and extracting information from the dataset [23,40]. Therefore, chemometrics helps to reduce the complexity of large chemical data sets, offering better understandings, simplifications, explanations, and accuracy in results.

In recent years, statistical techniques combined with traditional analytical techniques, as well as melissopalynology, have been proven successful in making decisions about differentiation criteria in the complex matrix of honey [1,5,15,21,22,40]. Attempts at assessing botanical or geographic origins are made based on the physicochemical and antioxidant properties of honeys or their chemical composition with the use of multivariate techniques. Especially, PCA, CA and LDA are the best-known and most used techniques in the classification of honeys with satisfactory results [5,18,20,21,27,28,29,38]. Some Spanish honeys from Canary Island (fennel, chestnut, retama, and tajinaste honeys) were correctly classified (95.1%) by PCA-LDA, choosing the physicochemical variables (EC, acidity, pH, color, proline, diastase, invertase, fructose, glucose, trehalose, and melezitose) [20]. The quality parameters such as moisture, EC and pH were the variables that better discriminated Uruguayan honeys, with more than 80% of the samples correctly classified according to their floral origin (pasture, *Citrus* and *Baccharis*) by PCA-LDA [35]. The research carried out by Tarapatskyy et al. [29] showed that the specific content of phenolic acids, minerals, proline, and sugar, in combination with chemometric analysis (PCA, CA, and LDA), can successfully differentiate Polish honey samples according to their botanical origin (lime, buckwheat, and pine honeydew), as a preliminary verification of samples before performing pollen analysis. The application of an LDA model succeeded in classifying the Italian unifloral honeys (acacia, orange, honeydew, chestnut, strawberry tree, sulla, eucalyptus, dandelion, and linden), as they greatly differed in the polyphenol content and color attributes due to their botanical origin [18]. Eight physicochemical parameters (L*, a*, total dissolved solids, salinity, moisture, free acidity, total acidity, and dissolved solids/total acidity ratio) were enough for classifying Egyptian honeys such as clover or citrus honey. LDA function is classified correctly at a rate higher than 90% [41]. Akbari et al. [23] provided a 97% classification rate with PCA-DA for Iranian honeys from thyme, jujube, coriander, barberry, acacia, and alfalfa. PCA and LDA identified as significant variables main pollen data and physicochemical variables (free acidity, reducing sugars, and moisture) to build a discriminatory model with a cumulative variance of 90%, and correctly classifying three different groups of Argentine honeys (*Eucalyptus*, *Salix humboldtiana*, and *Baccharis*) [40]. Other mathematical treatments covering PCA and LDA models included a sugars profile for the discrimination of honeys from Southern Italy (Calabria region) by the botanical origin [28]. Some antioxidant properties, minerals, and color had the highest discriminating power for cherry, apple, saffron, and wild bush honeys collected in India, with a successful classification of 100% by LDA-CA [38]. In the case of the samples from Northwest Spain, the variables that better contributed to the discrimination between chestnut and honeydew honeys with PCA-LDA (97.6% of samples) were moisture, diastase, CIELab coordinates for color, flavonoids, radical scavenging activity, Mg, Na, fructose, turanose, maltose, trehalose, and main pollen variables [21]. The variables with the greatest discriminatory power using LDA for unifloral honeys of *Citrus* and *Eucalyptus* from southern Spain were water activity and EC [19]. Chemometrics provided satisfactory results for the classification of honey samples covered by PGI *Miel de Galicia* of the present study. All the blackberry, eucalyptus and heather honeys were properly grouped. In the case of chestnut and honeydew honeys, some samples were misclassified (5 and 14, respectively). It should be highlighted that chestnut honey and honeydew honey presented similar fingerprints regarding the routine physicochemical parameters, only a slightly high EC and color in honeydew honeys are marked, but the natural variations for each type of honeys complicate a full differentiation [21]. Other analyses, such as sensorial analysis, the polyphenol or volatile profile, could contribute to the discrimination. Choosing the most suitable chemical compounds for the discrimination of honey samples based on botanical origin is a complicated task and requires long professional experience in analytical techniques and knowledge of the composition and origin of this complex matrix. Some studies demonstrated the strong relationship between the floral origin of honey with physicochemical parameters, the profile and quantity of bioactive compounds (polyphenols, minerals) and color attributes. Therefore, these variables can be used as a simple approach for the discrimination of floral origin and, at the same time, as a preliminary evaluation of the antioxidant properties of honey.

## 5. Conclusions

Chemometrics contributed to the discrimination of the botanical origin of honeys produced in Galicia (NW Spain). The statistical classification methods on a large number of honey samples from different floral origins were tested, including unifloral honeys collected in PGI *Miel de Galicia*. The variables with the greatest weight in the differentiation of honeys based on PCA were EC, TPC, TFC, and dominant pollens (*Eucalyptus*, *Erica*, *Rubus*, and *Castanea sativa*). LDA classified the honeys in the function of the botanical origin, simplifying the interpretation of data from samples of the same geographical area. LDA showed better results graphically in the differentiation of the honey groups than CA. However, the differentiation of honeydew honey with chestnut honey from Galicia can sometimes be difficult due to having similar qualities in pH, EC, enzyme content, TPC, and TFC. TPC and TFC were higher for dark-colored honey (chestnut, heather, and honeydew) compared to light-colored honey (blackberry and eucalyptus). Therefore, the application of multivariate techniques helps to characterize honeys according to their botanical origin linked to a differentiating quality and to the geographical territory.

## Figures and Tables

**Table 1:** Descriptive analysis of the families and pollen types found in more than 30% of honey samples (Rep.). Distribution of the samples according to the frequency classes (D: dominant pollen; A: accompanying pollen; I: important pollen; R: frequent pollen; P: minor pollen). Max.: maximum.

Family	Pollen Type	Rep.(%)	Mean	SD	Max.	D > 45	A (15–45)	I (3–15)	R (1–3)	P (0–1)
Rosaceae	*Rubus*	99.2	23.2	19.8	91.3	18.0	37.6	30.4	7.7	5.5
Fagaceae	*Castanea sativa*	98.6	42.9	24.8	92.5	45.6	38.1	9.7	3.6	1.7
Fabaceae	*Cytisus* type	97.8	5.3	6.6	48.6	0.3	6.6	45.9	29.0	16.0
Ericaceae	*Erica*	96.7	7.9	11.6	68.6	1.9	14.9	35.6	22.4	21.8
Myrtaceae	*Eucalyptus*	81.5	13.4	23.8	94.8	11.9	10.5	17.1	16.9	25.1
Fabaceae	*Trifolium* type	73.8	0.8	1.5	15.4	-	0.3	6.4	16.9	50.3
Fagaceae	*Quercus*	71.8	0.7	1.9	27.8	-	0.3	4.7	14.9	51.9
Boraginaceae	*Echium*	67.1	0.9	1.8	13.1	-	-	9.4	13.8	43.9
Salicaceae	*Salix*	59.7	0.7	2.3	27.4	-	0.8	4.1	10.5	44.2
Plantaginaceae	*Plantago*	53.6	0.2	0.6	7.4	-	-	0.6	4.4	48.6
Poaceae	Poaceae	53.3	0.2	0.5	3.3	-	-	0.8	5.8	46.7
Rosaceae	*Crataegus monogyna* type	44.5	0.3	1.1	17.5	-	0.3	1.9	6.4	35.9
Rosaceae	*Prunus* type	42.0	0.2	0.4	3.9	-	-	0.8	2.5	38.7
Apiaceae	*Conium maculatum* type	41.4	0.3	0.7	7.0	-	-	1.4	5.2	34.8
Brassicaceae	*Brassica* type	40.1	0.2	0.5	5.5	-	-	0.6	3.3	36.2
Rhamnaceae	*Frangula alnus*	35.9	0.4	1.8	21.8	-	0.6	1.4	6.4	27.6
Campanulaceae	*Campanula* type	35.1	0.2	0.4	3.9	-	-	0.6	3.3	31.2
Resedaceae	*Sesamoides*	34.3	0.2	0.9	16.0	-	0.3	-	3.3	30.7
Scrophulariaceae	*Scrophularia* type	32.3	0.1	0.2	2.9	-	-	-	1.1	31.2

**Table 2:** Descriptive analysis of the physicochemical parameters determined in the set of honey samples. SD: standard deviation, EC: electrical conductivity; TPC: total phenol content; TFC: total flavonoid content.

	Mean	SD	Minimum	Maximum
Moisture (%)	17.74	1.02	14.40	21.20
pH	4.24	0.35	3.29	5.15
EC (mS/cm)	0.76	0.28	0.22	1.65
Diastase content	21.31	8.79	6.14	44.04
TPC (mg/100 g)	116.43	44.39	33.91	254.50
TFC (mg/100 g)	6.72	2.72	1.28	16.70
Color (mm)	105	30	36	150

**Table 3:** Results of components extracted and weights of pollen and physicochemical variables by PCA. EC: electrical conductivity; TPC: total phenol content; TFC: total flavonoid content.

Components	C1	C2	C3	C4
Eigenvalue	4.03	1.88	1.29	1.18
Variance (%)	36.63	17.08	11.73	10.69
Cumulative variance (%)	36.63	53.71	65.45	76.14
Component weights of pollen variables				
*Rubus*	−0.02	−0.38	0.72	0.07
*Castanea sativa*	−0.30	−0.08	−0.18	−0.54
*Erica*	−0.01	0.60	0.23	0.11
*Eucalyptus*	0.31	0.06	−0.54	0.34
Component weights of physicochemical variables				
Moisture	−0.03	0.41	0.06	−0.55
pH	−0.29	−0.34	−0.15	0.21
EC	−0.44	−0.10	−0.19	−0.03
Diastase content	−0.31	−0.15	−0.19	−0.20
TPC	−0.43	0.09	0.02	0.28
TFC	−0.42	0.17	0.08	0.22
Color	−0.29	0.37	0.03	0.27

**Table 4:** Results of the statistics according to LDA. DF: degrees of freedom.

Discriminant Function	EigenValue	Relative Percentage	Canonical Correlation	Wilks Lambda	Chi-Square	DF	*p*
1	3.98	37.27	0.89	0.01	1777.24	55	<0.001
2	3.48	32.54	0.88	0.03	1211.01	40	<0.001
3	2.29	21.42	0.83	0.14	682.5	27	<0.001
4	0.7	6.52	0.64	0.47	262.65	16	<0.001
5	0.24	2.26	0.44	0.81	76.31	7	<0.001

**Table 5:** Number of samples and correct percentage of classification (in brackets) by honey type according to the LDA.

			Predicted Honey Type (%)				
Honey Type	*n*	Chestnut	Blackberry	Eucalyptus	Heather	Honeydew	Multifloral
Chestnut	52	47 (90.4)	0	-	-	2 (3.8)	3 (5.8)
Blackberry	56	0	56 (100)	-	-	0	0
Eucalyptus	33	0	0	33 (100)	0	0	0
Heather	36	0	0	0	36 (100)	0	0
Honeydew	53	7 (13.2)	5 (9.4)	0	0	39 (73.6)	2 (3.8)
Multifloral	132	3 (2.3)	4 (3.0)	6 (4.5)	3 (2.3)	2 (1.5)	114 (86.4)

**Table 6:** Descriptive analysis of the botanical and physicochemical characteristics by honey type based on LDA. * The numbers show significant differences in the means for each honey type (1: chestnut, 2: blackberry, 3: eucalyptus, 4: heather, 5: honeydew and 6: multifloral) according to the Bonferroni test (*p* < 0.05). SD: standard deviation, PG/g: number of pollen grains per gram of honey; EC: electrical conductivity; TPC: total phenol content; TFC: total flavonoid content.

	Mean	SD	Lower Limit than 95%	Upper Limit than 95%	ANOVA *
***Chestnut honey*** (*n* = 57)					
*Rubus* (%)	12.6	6.7	10.8	14.4	2, 3, 5, 6
*Castanea sativa* (%)	76.8	8.0	74.6	78.9	2, 3, 4, 5, 6
*Erica* (%)	4.0	3.7	3.0	5.0	4
*Eucalyptus* (%)	1.3	2.5	0.6	1.9	3, 6
PG/g	30,239	23,505	23,885	36,593	3, 4, 6
Moisture (%)	18.2	1.0	17.9	18.5	2, 3, 5, 6
pH	4.5	0.4	4.4	4.6	2, 3, 4, 6
EC (mS/cm)	1.02	0.21	0.97	1.08	2, 3, 4, 6
Diastase content	23.5	7.2	21.6	25.4	3, 5
TPC (mg/100 g)	122.8	29.4	115.0	130.6	2, 3, 5
TFC (mg/100 g)	8.2	2.2	7.6	8.8	2, 3, 5, 6
Color (mm Pfund)	128	24	122	135	2, 3, 6
***Blackberry honey*** (*n* = 65)					
*Rubus* (%)	56.7	11.4	53.9	59.5	1, 3, 4, 5, 6
*Castanea sativa* (%)	26.6	13.9	23.1	30.0	1, 3, 5, 6
*Erica* (%)	3.3	4.5	2.1	4.4	4, 6
*Eucalyptus* (%)	1.6	2.5	0.9	2.2	3, 6
PG/g	26,571	29,847	18,723	34,418	4
Moisture (%)	17.4	1.0	17.1	17.6	1, 4
pH	4.3	0.3	4.2	4.4	1, 3, 4, 5, 6
EC (mS/cm)	0.69	0.25	0.63	0.75	1, 3, 5
Diastase content	19.7	7.0	18.0	21.5	3, 5
TPC (mg/100 g)	94.7	31.7	86.8	102.5	1, 4, 5
TFC (mg/100 g)	6.1	2.1	5.6	6.7	1, 3, 4, 5, 6
Color (mm Pfund)	96	30	89	104	1, 3, 4, 5
***Eucalyptus honey*** (*n* = 39)					
*Rubus* (%)	2.2	2.6	1.3	3.0	1, 2, 4, 5, 6
*Castanea sativa* (%)	8.3	7.9	5.7	10.9	1, 2, 4, 5, 6
*Erica* (%)	3.0	3.3	1.9	4.0	4, 6
*Eucalyptus* (%)	73.8	11.4	70.1	77.5	1, 2, 4, 5, 6
PG/g	16,371	10,946	12,126	20,615	1
Moisture (%)	17.4	0.9	17.1	17.7	1, 4
pH	4.1	0.3	4.0	4.2	1, 2, 5
EC (mS/cm)	0.51	0.10	0.48	0.54	1, 2, 4, 5, 6
Diastase content	14.6	7.0	12.4	16.9	1, 2, 4, 5, 6
TPC (mg/100 g)	83.7	38.2	71.3	96.1	1, 4, 5, 6
TFC (mg/100 g)	4.6	1.2	4.2	5.0	1, 2, 4, 5
Color (mm Pfund)	77	19	71	83	1, 2, 4, 5, 6
***Heather honey*** (*n* = 39)					
*Rubus* (%)	10.9	8.7	8.1	13.7	2, 3, 5, 6
*Castanea sativa* (%)	28.4	14.7	23.6	33.1	1, 3, 5, 6
*Erica* (%)	36.7	11.0	33.1	40.3	1, 2, 3, 5, 6
*Eucalyptus* (%)	5.6	9.5	2.6	8.7	3, 6
PG/g	11,045	11,028	7420	14,670	1, 2
Moisture (%)	18.5	1.2	18.1	18.9	2, 3, 5, 6
pH	4.0	0.2	3.9	4.1	1, 2, 5
EC (mS/cm)	0.68	0.17	0.62	0.73	1, 3, 5
Diastase content	20.1	8.2	17.4	22.8	3, 5
TPC (mg/100 g)	143.1	49.6	127.0	159.2	2, 3, 6
TFC (mg/100 g)	7.3	2.0	6.7	8.0	2, 3, 5, 6
Color (mm Pfund)	117	21	110	124	2, 3, 5, 6
***Honeydew honey*** (*n* = 43)					
*Rubus* (%)	26.9	12.7	23.0	30.8	1, 2, 3, 4, 6
*Castanea sativa* (%)	53.6	19.1	47.7	59.4	1, 2, 3, 4
*Erica* (%)	3.5	3.6	2.4	4.6	4
*Eucalyptus* (%)	1.0	2.3	0.3	1.7	3, 6
PG/g	18,531	14,365	13,352	23,710	
Moisture (%)	17.4	0.9	17.1	17.6	1, 4
pH	4.5	0.2	4.4	4.5	2, 3, 4, 6
EC (mS/cm)	1.14	0.20	1.07	1.20	2, 3, 4, 6
Diastase content	29.4	7.9	27.0	31.8	1, 2, 3, 4, 6
TPC (mg/100 g)	166.2	39.2	154.1	178.3	1, 2, 3, 6
TFC (mg/100 g)	11.2	2.2	10.5	11.8	1, 2, 3, 4, 6
Color (mm Pfund)	142	15	137	146	2, 3, 4, 6
***Multifloral honey*** (*n* = 119)					
*Rubus* (%)	19.5	11.7	17.4	21.6	1, 2, 3, 4, 5
*Castanea sativa* (%)	47.8	17.0	44.7	50.9	1, 2, 3, 4
*Erica* (%)	6.2	6.1	5.1	7.3	2, 3, 4
*Eucalyptus* (%)	12.8	14.8	10.1	15.5	1, 2, 3, 4, 5
PG/g	18,153	18,868	14,390	21,916	1
Moisture (%)	17.7	0.8	17.6	17.9	1, 4
pH	4.1	0.3	4.1	4.2	1, 2, 5
EC (mS/cm)	0.66	0.19	0.62	0.69	1, 3, 5
Diastase content	20.8	9.1	19.1	22.4	3, 5
TPC (mg/100 g)	109.3	37.9	102.4	116.2	3, 4, 5
TFC (mg/100 g)	5.2	1.4	5.0	5.5	1, 2, 4, 5
Color (mm Pfund)	91	21	87	94	1, 3, 4, 5

## Data Availability

Data is contained within the article.
